# Calendar month seasonality of HbA1c and its phase relationship with climatological temperature in 633,838 clinical measurements

**DOI:** 10.3389/fendo.2026.1990386

**Published:** 2026-10-07

**Authors:** Kecheng Li, Fei Xia

**Affiliations:** Laboratory Department of Ruian People’s Hospital, Zhejiang, Wenzhou, China

**Keywords:** ambient temperature, diabetes mellitus, glycated haemoglobin, lagged association, seasonal variation, time-series

## Abstract

**Objective:**

HbA1c reflects glucose exposure over the preceding 8–12 weeks. We characterised the lagged seasonal association between monthly climatological temperature normals and HbA1c in a 12-year hospital cohort and examined whether a similar pattern was present in patient-level records.

**Research design and methods:**

We analysed 633,838 HbA1c measurements (387,678 patients; 633,737 with complete covariates) from a Chinese tertiary hospital (2014–2025). Cross-correlation was assessed for lags of 0–6 months using monthly HbA1c means and fixed monthly climatological temperature normals. Patient-level distributed-lag regression with cluster-robust standard errors and generalised estimating equations was used as a complementary analysis.

**Results:**

HbA1c was inversely correlated with the climatological temperature series most strongly at a 2-month lag (r = −0.297, P < 0.001) rather than concurrently (r = −0.127, P = 0.14). Pooled by calendar month, the lag-2 seasonal association was strong (r = −0.977, R² = 0.955), with a seasonal HbA1c amplitude of 0.235% (2.6 mmol/mol; peak March, trough September), strongest under age 60 and attenuated during COVID-19. Patient-level regression showed a similar lag pattern, with the largest coefficient at lag 2 (β = −0.0088% per °C; 95% CI −0.0093 to −0.0082; P < 0.001), and the individual-level partial R² was 0.15%.

**Conclusions:**

The study identified a reproducible seasonal HbA1c pattern that aligned most strongly with the 2-month-shifted climatological temperature series, with significant correspondence also at 1 and 3 months. Because the exposure was a fixed calendar-month normal, the findings should be interpreted as a lagged seasonal association rather than evidence of a causal temperature effect or a specific 2-month biological delay. The small individual-level partial R² (0.15%) also argues against applying the population amplitude directly to individual treatment decisions. Future research should evaluate whether seasonal contextualisation can optimise laboratory monitoring and re-testing strategies near diagnostic thresholds.

## Introduction

Glycated haemoglobin (HbA1c) is the gold-standard biomarker for long-term glycaemic monitoring in diabetes mellitus, and its clinical interpretation rests on the assumption that it reflects mean blood glucose concentrations over the preceding 8–12 weeks (1). This time window is determined by the lifespan of circulating erythrocytes and the kinetics of non-enzymatic haemoglobin glycation — a process in which glucose reacts spontaneously with terminal amino groups on haemoglobin chains at a rate proportional to ambient glucose concentration. Crucially, the glycation reaction is itself temperature-sensitive: higher temperatures accelerate non-enzymatic glycation *in vitro*, whilst lower temperatures reduce glycation rates (2, 3).This suggests that, through both direct biochemical effects and the indirect mediation of physical activity, dietary behaviour, and vitamin D metabolism, environmental temperature should leave a measurable imprint on HbA1c — but with a lag corresponding to the biological integration window of approximately 2 months.

This raises a clinically relevant possibility. If HbA1c measured in February reflects the physiological conditions of December and January — typically the coldest months of the year — then population mean HbA1c may be higher in winter than in summer on account of seasonal physiology rather than any change in diabetes management. Such “seasonal HbA1c variation” creates potential artefacts at the population level: comparisons of aggregate results across seasons may attribute seasonal fluctuation to treatment success or failure (4, 5). Epidemiological studies that use HbA1c as a primary outcome without adjusting for temperature or season may reach biased conclusions.

Despite this physiological logic, the literature on temperature–HbA1c associations is surprisingly inconsistent, in part because most studies use concurrent (lag 0) temperature rather than lagged exposures that correspond to the HbA1c integration window (6, 7). Studies from Japan, Taiwan, Vietnam, and several Western countries have documented seasonal HbA1c variation and temperature-related metabolic fluctuations (8),but systematic lag analysis covering lags 0–6 months has rarely been performed in Chinese populations (9, 10), and never in a dataset spanning 12 years with over 630,000 measurements.

We address these gaps using 633,838 HbA1c measurements from a large Chinese tertiary hospital (2014–2025). Our objectives were: (1) to determine at which lag temperature most strongly predicts HbA1c using systematic cross-correlation analysis; (2) to quantify the magnitude of temperature-attributable seasonal HbA1c variation; (3)to test whether the lag-2 association persists after adjustment for harmonic seasonal terms; and (4) to examine whether age and the COVID-19 pandemic modified the temperature–HbA1c relationship.

## Research design and methods

### Study design and data source

This retrospective time-series study used routinely collected laboratory data from a Chinese tertiary hospital. All HbA1c measurements entered into the laboratory information system between January 2014 and December 2025 were extracted, including a unique patient identifier, test date, sex, age, and care setting. HbA1c values outside the plausible clinical range (3.0–20.0%) were excluded. To reduce instability in monthly estimates, monthly time strata with fewer than 500 tests were excluded; eight month-year strata met this prespecified criterion (February 2014, February 2015, February 2016, February 2020, January 2021, February 2021, April 2022, and December 2022). Most of these months coincided with the Spring Festival period or periods of acute COVID-19 control, when routine outpatient screening and testing volumes were temporarily reduced.

### Temperature data

Monthly mean ambient temperature data were obtained from publicly available climatological records (1991–2020 normals) for Wenzhou, Zhejiang Province, China, representing multi-year monthly normal values: January 8.2 °C, February 9.4 °C, March 12.8 °C, April 17.6 °C, May 22.1 °C, June 25.5 °C, July 28.9 °C, August 28.4 °C, September 25.2 °C, October 20.8 °C, November 15.9 °C, December 10.4 °C. These climatological normals were used for all analyses.

### Statistical analysis

Monthly mean HbA1c was calculated across 136 eligible monthly time points. Temperature variables at lags 0–6 months (temp_lag0 to temp_lag6) were derived by shifting the fixed monthly climatological normal series. Cross-correlation analysis computed Pearson r and Spearman ρ with 95% confidence intervals (Fisher z-transformation) for each lag. Newey–West robust standard errors (lag bandwidth = 6) were used in regression models to account for autocorrelation and heteroscedasticity. Three regression models were estimated: Model A (temperature lag 2 only), Model B (temperature lag 2 + harmonic seasonal terms sin(2πt/12) and cos(2πt/12)), and Model C (harmonic terms only, for partial R² calculation). Because the temperature values were deterministic functions of calendar month, the harmonic terms were interpreted as adjustment for seasonal structure rather than as a means of identifying an independent temperature effect. Seasonal analysis pooled observations by calendar month across all years. Age-stratified analyses were performed for four age groups: <45, 45–59, 60–74, and ≥75 years. Period-stratified correlations were computed for pre-COVID (2014–2019), COVID period (2020–2022), and post-COVID (2023–2025) subsets. All analyses were performed in R 4.3 using sandwich, lmtest, and ggplot2 packages.

### Patient-level distributed-lag modelling

The analyses above operate on monthly aggregates and are therefore susceptible to ecological bias: associations estimated on group means need not hold, in magnitude or even direction, at the level of the individual patient. To examine whether the lag pattern was also present in individual records, we re-analysed 633,737 of the 633,838 eligible test records (99.98%; the remaining 101 records were excluded for missing age, sex, or care-setting data required for covariate adjustment), corresponding to 387,598 of the 387,678 unique patients, without monthly aggregation. As in the ecological analysis above, temperature at each lag was assigned from the multi-year monthly climatological normal for the corresponding calendar month, so every patient tested in the same calendar month received an identical temperature-at-lag-l value. For each lag l (0–6 months), ordinary least-squares regression of HbA1c on temperature-at-lag-l was fitted with adjustment for age, sex, and care setting, using cluster-robust standard errors grouped by patient identifier to account for repeated testing within the same individual. A mutually adjusted seven-lag model was fitted as an exploratory sensitivity analysis, with variance inflation factors used to quantify collinearity. A GEE with an exchangeable working correlation structure was also fitted amongst patients with repeated measurements. These analyses were intended to assess consistency of the lag pattern, not to remove the underlying calendar-month exposure limitation.

## Results

### Study population and temporal characteristics

After applying the inclusion criteria, 633,838 HbA1c measurements from 387,678 unique patients were included across 136 monthly time points. The overall mean HbA1c was 6.63 ± 1.63% (49 mmol/mol), with a median of 6.10% (43 mmol/mol; IQR 5.5–7.4%). Descriptive characteristics by study period are presented in **Table 1**, and the full time-series co-variation of monthly mean HbA1c and the climatological temperature series is shown in **Supplementary Figure S1**. Monthly mean HbA1c showed a seasonal pattern, with higher values observed during late winter and spring, reaching a pooled peak in March (6.735%), and lower values in September (6.500%). The pooled seasonal amplitude across the 12-year study period was 0.235%. Period-specific seasonal amplitudes ranged from 0.38% to 0.39% across the three study periods (**Table 1**).

**Table 1:** Descriptive characteristics and seasonal HbA1c pattern by study period.

Characteristic	2014–2019 (n=285,636)	2020–2022 (n=145,989)	2023–2025 (n=202,213)	Overall (n=633,838)
Tests per year, median [range]	49,736 [28k–74k]	48,663 [19k–64k]	67,405 [64k–71k]	58,485 [19k–71k]
Mean HbA1c (%), mean ± SD	6.81 ± 1.66	6.59 ± 1.60	6.41 ± 1.59	6.63 ± 1.63
Median HbA1c (%), median [IQR]	6.25 [5.6–7.7]	6.09 [5.5–7.2]	5.90 [5.3–7.1]	6.10 [5.5–7.4]
HbA1c ≤7.0%, n (%)	197,082 (69.0)	106,270 (72.8)	152,544 (75.4)	455,896 (71.9)
HbA1c >9.0%, n (%)	27,993 (9.8)	11,679 (8.0)	14,558 (7.2)	54,230 (8.6)
Seasonal HbA1c amplitude*	0.39%	0.38%	0.38%	0.235% (pooled)
Peak calendar month	March–April	March–April	January–March	March
Trough calendar month	September	September	September	September

*Period-specific amplitudes represent intra-period calendar-month ranges, whereas the overall pooled amplitude (0.235%) reflects the smoothed 12-year average calendar-month contrast; the two are not directly comparable and neither figure is more “correct” than the other.

### Cross-correlation analysis

**Supplementary Table S1** and **Figure 1a** summarise the cross-correlation between the fixed climatological temperature series and monthly mean HbA1c. The correlation was strongest at lag 2 months (r = −0.297, 95% CI −0.444 to −0.134; P < 0.001), with significant correlations also observed at lags 1 and 3 months. Because the lag-2 and lag-3 coefficients were not formally compared, the data are better interpreted as showing a 1–3 month window of strongest seasonal correspondence rather than a uniquely established 2-month biological delay.

**Figure 1:**
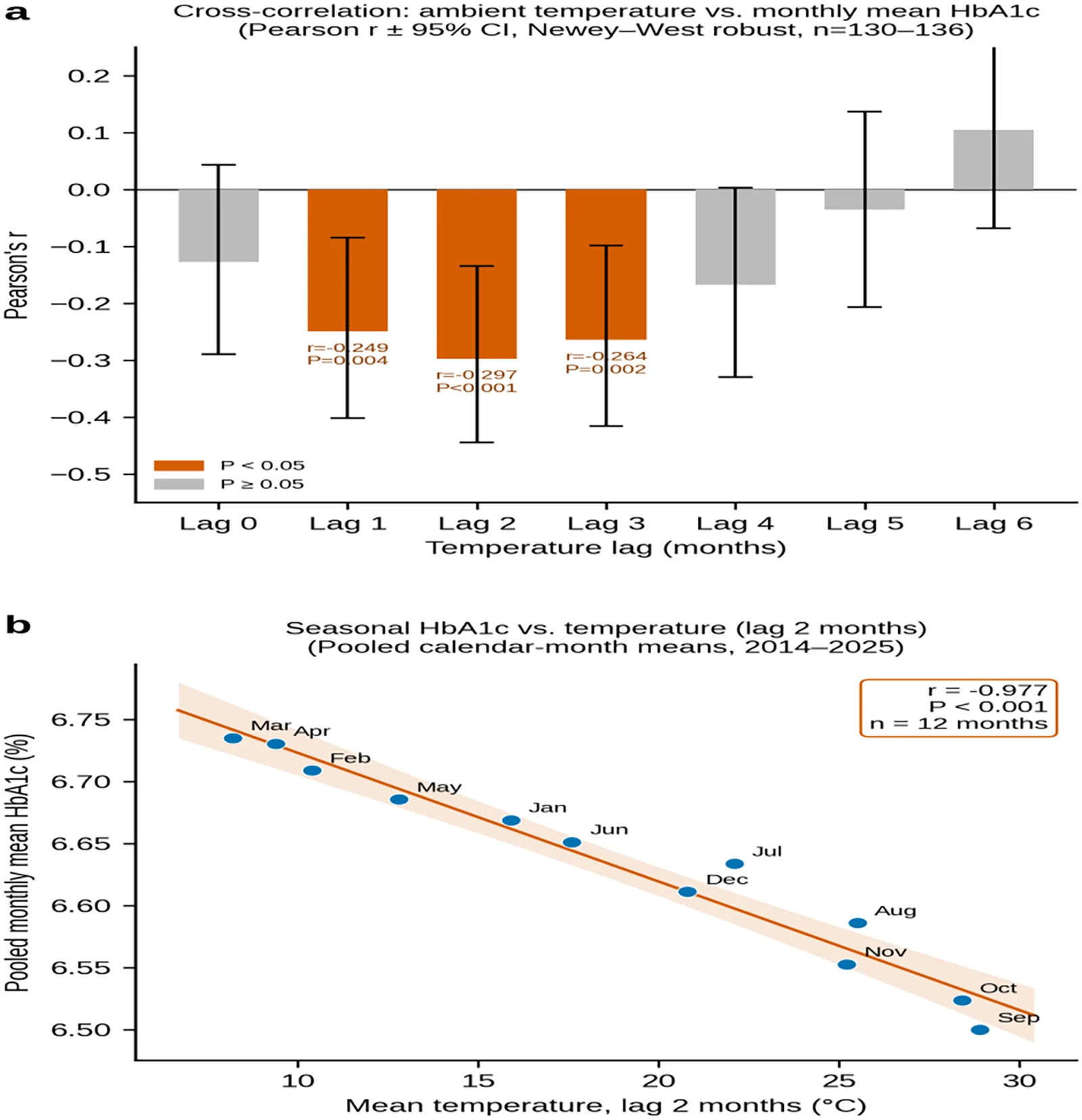
Cross-correlation and seasonal pattern. **(a)** Cross-correlation coefficients (Pearson r with 95% CI) for the climatological temperature series at lags 0–6 months versus monthly mean HbA1c (full time series, n = 130–136). Red bars: statistically significant (P < 0.05); grey bars: non-significant. Dashed horizontal lines indicate the approximate 95% confidence bounds for Pearson r based on Fisher z-transformation. **(b)** Scatter plot of pooled calendar-month mean HbA1c versus temperature lagged 2 months, with linear regression line and 95% CI. Each point represents one calendar month (Jan–Dec); month abbreviations labelled. r = −0.977, P < 0.001.

### Seasonal analysis and regression models

Seasonal analysis pooling observations by calendar month across the 12-year study period (n = 12 calendar-month strata) showed a strong inverse correlation between the lag-2 climatological temperature series and pooled monthly mean HbA1c (Pearson r = −0.977, 95% CI −0.994 to −0.917; P < 0.001; **Figure 1b**). The highest pooled mean HbA1c was observed in March (6.735%), whereas the lowest was observed in September (6.500%), corresponding to a seasonal amplitude of 0.235%. This correlation reflects a close correspondence between two calendar-month patterns and should not be interpreted as an estimate of an independent temperature effect.

In regression analyses, each 1 °C increase in the climatological temperature series 2 months prior was associated with a decrease in mean HbA1c of 0.00890% (95% CI −0.014 to −0.004; P < 0.001; Model A R² = 0.088). After adjustment for harmonic seasonal terms, the coefficient remained significant (β = −0.00862% per °C; P < 0.001; Model B R² = 0.103). Given the deterministic relationship between the exposure and calendar month, these models quantify seasonal correspondence rather than an independently identifiable causal temperature effect. Full regression results are provided in **Supplementary Table S2**.

### Age-stratified and period-stratified analyses

Age-stratified analyses showed variation in the lag-2 temperature–HbA1c correlation across age groups (**Supplementary Figure S2**). The correlation coefficients were −0.944 in patients aged <45 years, −0.972 in those aged 45–59 years, −0.846 in those aged 60–74 years, and −0.716 in those aged ≥75 years (all P < 0.01).

The lag-2 temperature–HbA1c correlation also varied across study periods. A significant inverse correlation was observed during the pre-COVID period (2014–2019; r = −0.566, P < 0.001) and the post-COVID period (2023–2025; r = −0.761, P < 0.001), whereas the association was not statistically significant during the COVID period (2020–2022; r = −0.229, P = 0.241).

### Patient-level assessment of the seasonal HbA1c pattern

The analyses above were based on aggregated monthly data. To evaluate the lagged temperature–HbA1c association at the individual-record level, we analysed 633,737 eligible HbA1c measurements without monthly aggregation, adjusting for age, sex, and care setting, with standard errors clustered by patient identifier. The lag-response pattern was similar to that observed in the monthly analysis (**Table 2**; **Figure 2a**). The association increased from lag 0 (β = −0.0029, 95% CI −0.0035 to −0.0024; P < 0.001) to a maximum at lag 2 months (β = −0.0088, 95% CI −0.0093 to −0.0082; P < 0.001), and declined thereafter, reaching a positive coefficient at lag 6 months (β = 0.0029, 95% CI 0.0024 to 0.0035; P < 0.001).

**Table 2:** Patient-level, covariate-adjusted, cluster-robust distributed-lag regression of HbA1c on ambient temperature (n = 633,737 records; 387,598 patients).

Temperature lag (months)	β (%HbA1c per °C)	95% CI	P value
Lag 0	-0.0029	-0.0035 to -0.0024	<0.001
Lag 1	-0.0068	-0.0074 to -0.0062	<0.001
Lag 2 (peak)	-0.0088	-0.0093 to -0.0082	<0.001
Lag 3	-0.0083	-0.0088 to -0.0077	<0.001
Lag 4	-0.0057	-0.0062 to -0.0051	<0.001
Lag 5	-0.0016	-0.0021 to -0.0010	<0.001
Lag 6	0.0029	0.0024 to 0.0035	<0.001

Each row is a separate model adjusting for age, sex, and care setting, with standard errors clustered by patient identifier.

**Figure 2:**
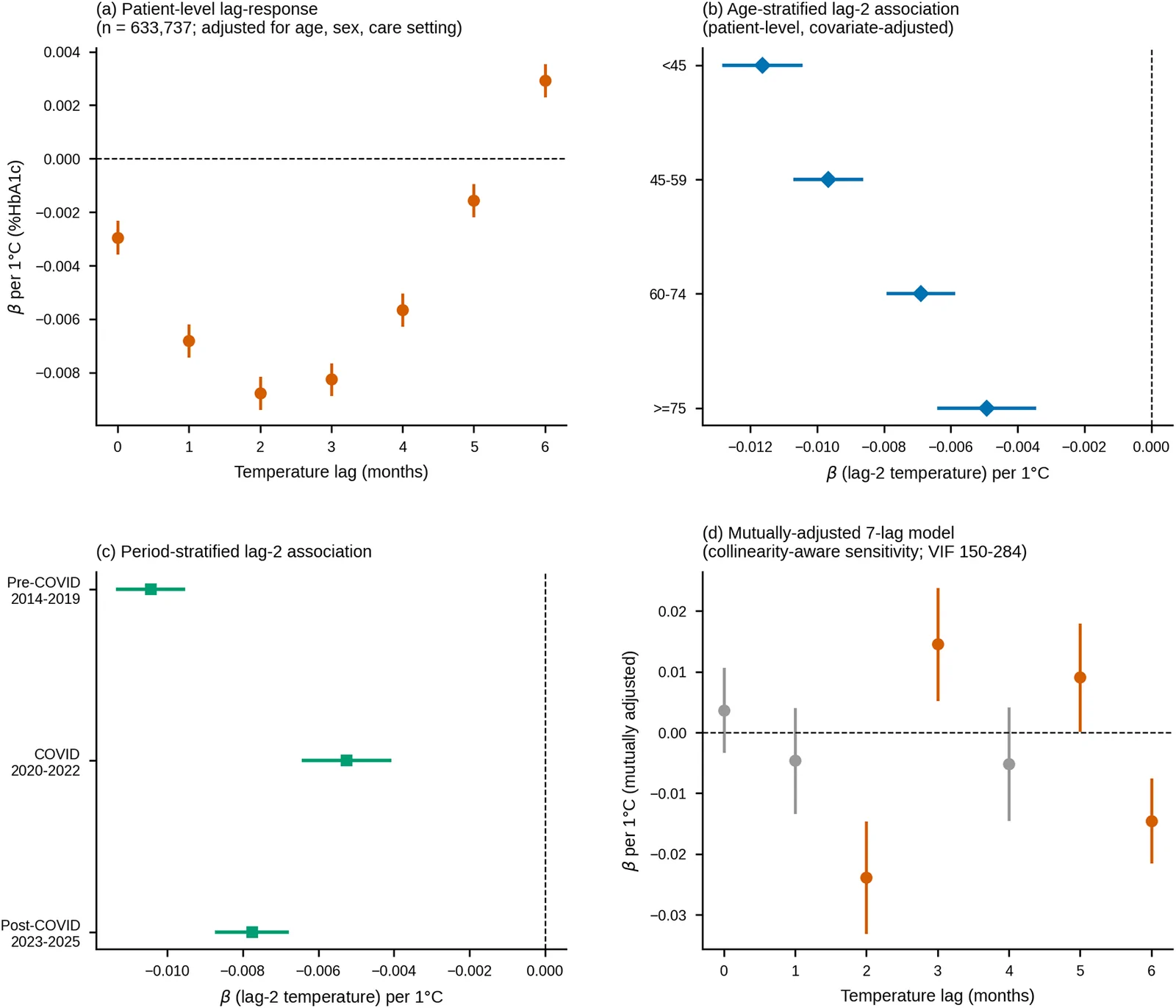
Patient-level assessment of the lag structure. **(a)** Lag-response curve from seven separate patient-level models (β per 1 °C, 95% CI); red markers indicate P < 0.05. **(b)** Age-stratified lag-2 association. **(c)** Period-stratified lag-2 association. **(d)** Mutually-adjusted seven-lag model (collinearity-aware sensitivity; VIF 150–284).

Because lagged temperature variables were highly correlated, a mutually adjusted model including all seven lag terms was additionally fitted as an exploratory sensitivity analysis. Variance inflation factors ranged from 150 to 284, indicating severe collinearity. Although lag-2 (β = −0.0238) and lag-6 (β = −0.0145) remained nominally significant in this model, these coefficients were not interpreted as independent lag effects because of the high collinearity (**Figure 2d**). A generalised estimating equation with an exchangeable working correlation structure, fitted amongst the 337,493 records from 91,354 patients with repeated measurements, yielded a similar lag-2 association (β = −0.0067% per °C; P < 0.001).

Covariate-adjusted subgroup analyses showed consistent lag-2 associations across age groups and study periods (**Figures 2b, c**). The lag-2 coefficient decreased with increasing age (<45 years: β = −0.0116, 95% CI −0.0128 to −0.0105; 45–59 years: β = −0.0097; 60–74 years: β = −0.0069; ≥75 years: β = −0.0049, 95% CI −0.0060 to −0.0038; all P < 0.001). The lag-2 association was smaller during the COVID-19 period (β = −0.0053) than during the pre-COVID period (β = −0.0104) and remained attenuated in the post-COVID period (β = −0.0078; all P < 0.001).

The difference between aggregated and individual-record model fit was descriptive. The pooled monthly regression of mean HbA1c on lag-2 temperature yielded an R² of 0.955 (n = 12 calendar-month strata). At the individual-record level, lag-2 temperature increased the model R² from 0.0390 to 0.0404, corresponding to a partial R² of 0.0015 (0.15%) after adjustment for age, sex, and care setting. Because the exposure was identical for patients tested in the same calendar month, this analysis does not remove the underlying ecological and calendar-time limitations.

## Discussion

### Interpretation of the seasonal pattern

The main finding is the stability of the HbA1c seasonal pattern across a large clinical dataset. Similar seasonal variation has been reported in several countries (11), although the peak and trough months differ across settings (12). Studies from Singapore and other regions also indicate that festive periods, diet and activity may contribute to the annual cycle, even where temperature variation is limited (13–15). Our results should therefore be understood as a calendar month pattern with a parallel phase relationship to climatological temperature.

### Why the apparent 2 month lag should be interpreted cautiously

The multi-lag analysis showed that the HbA1c seasonal pattern aligned most strongly with the climatological temperature series shifted by 2 months, whilst significant correspondence was also present at lags 1 and 3 months (**Supplementary Table S1**). This pattern is compatible with the 8–12 week integration window of HbA1c, in which the dominant fraction of circulating haemoglobin at any given moment has been exposed to the glycation environment of approximately 5–10 weeks prior. The apparent 2-month optimum, however, is not equivalent to a demonstrated 2-month physiological delay. Because the temperature exposure repeats the same twelve monthly values in every year, shifting the series changes only the phase of a fixed annual cycle, and the present data therefore cannot distinguish a biological delay from a phase relationship between recurring calendar-month patterns. The similar association at three months further weakens the case for a single biological lag, and the non-significant concurrent (lag 0) correlation (r = −0.127, P = 0.140) is equally consistent with either interpretation.

The mechanistic account is similarly constrained. Because haemoglobin glycation takes place at a tightly regulated core temperature of approximately 37 °C, the *in vitro* comparison between 30 °C and 37 °C cited earlier (3) has uncertain bearing on normothermic individuals. More plausible explanations therefore lie in behavioural and metabolic pathways, including seasonal variation in diet, physical activity, respiratory infection, and infection-related changes in erythrocyte turnover (16). Observational studies in other populations have linked ambient temperature to HbA1c (8, 16), which lends biological plausibility but does not disentangle temperature from calendar month in the present analysis.

### Clinical and population implications

The observed seasonal amplitude is a property of the population of tests in this hospital and should not be converted into an individual correction factor. Current American Diabetes Association guidelines categorise HbA1c <7.0% (53 mmol/mol) as controlled and ≥8.0% (64 mmol/mol) as poorly controlled (1), but applying a population-average seasonal shift to an individual measurement would itself constitute an ecological inference, and this study did not evaluate whether seasonal variation changes treatment decisions. The amplitude should also be considered relative to within-patient biological variation and analytical imprecision (17, 18), both of which are of comparable or greater magnitude. The more defensible applications are therefore population surveillance, institutional benchmarking, and quality indicators, in which the timing of testing may vary systematically across months and the monthly average may shift without any true change in glycaemic control. A seasonal adjustment may be considered in such settings only after validation in an independent dataset.

### Age as a modifier of the temperature–HbA1c relationship

The attenuation of the lag-2 correlation with age warrants specific discussion. Patients under 60, whose glycaemia may be more responsive to physiological and behavioural changes, showed the strongest seasonal coupling (r = −0.944 in <45 years; r = −0.972 in 45–59 years). Patients aged 60 and older showed a materially weaker association (r = −0.846 and −0.716, respectively), most blunted in those ≥75 years.

### Assessing the ecological limitation through patient-level regression

Monthly time-series correlation analyses are inherently ecological: an association estimated across group means need not hold for individual patients, and its apparent strength can be substantially inflated by the averaging process itself — a phenomenon formalised as the ecological-correlation paradox (17).Distributed-lag non-linear models (DLNM) were developed specifically to characterise delayed, non-linear exposure–response relationships at fine temporal resolution (19, 20), but because the present exposure series is a repeating climatological normal rather than a genuinely time-varying daily record, a full DLNM cross-basis was not separately identifiable from calendar month. We therefore implemented the individual-record analogue that this data structure supports — patient-level, covariate-adjusted, cluster-robust distributed-lag regression on all 633,737 original records — as a complementary sensitivity analysis. It showed a similar lag pattern, but because every patient tested in the same calendar month received an identical exposure value, it did not create within-month exposure contrast and did not remove the underlying ecological limitation.

Consistent with this interpretation, the pooled monthly R² (0.955) and the individual-level partial R² (0.0015) derive from models with different units of analysis and should not be compared or expressed as a ratio. The practical implication is that the observed seasonal signal is more relevant to aggregate monitoring and longitudinal laboratory interpretation than to individual correction of HbA1c. Recent studies converge on the same seasonal signal in independent populations: HbA1c shows consistent winter/spring elevation in adolescents with type 1 diabetes (21) and in mixed diabetic and non-diabetic outpatient cohorts (22); admission glucose in acute ischaemic stroke likewise varies with seasonal temperature (23); and pandemic-era disruption of routine seasonal HbA1c variation has been independently documented in a Taiwanese cohort (24), mirroring the COVID-period attenuation observed here.

### Future research

A stronger study would link each HbA1c measurement to observed year specific meteorological data and model temperature together with calendar season, long term trend and relevant time varying covariates. Such a design would permit more appropriate assessment of lag structure and clustering at the year month level. Where repeated measurements are available, within patient analyses could then assess whether seasonal associations persist after controlling for stable patient characteristics. External validation in other Chinese regions and climates would also be important. Ultimately, future research should evaluate whether seasonal adjustment models can be incorporated into clinical decision tools to reduce misclassification or unnecessary treatment changes near diagnostic and treatment thresholds.

### Limitations

Several limitations should be acknowledged. First, this is an observational study, and the observed lagged association cannot establish causality. Second, the temperature exposure was based on fixed multi-year monthly climatological normals rather than year-specific daily observations. Consequently, temperature is inseparable from calendar month in these data, and the findings cannot distinguish temperature from other seasonal factors such as diet, physical activity, holidays, healthcare-seeking behaviour, case mix, or infection-related changes. Third, the eight excluded month-years were selected because monthly testing volumes were below 500; although this improved temporal stability, exclusion may have introduced selection related to unusual service conditions. Fourth, individual temperature exposure, including indoor climate control, occupation, and residential environment, was not available (18, 25). Fifth, the study was conducted at a single centre and may not generalise to other climates or healthcare settings. Sixth, the cross-correlation analysis did not formally detrend or pre-whiten the series, so long-term HbA1c trends may have contributed to the lag correlations. Seventh, the high collinearity amongst lagged temperature variables means that the mutually adjusted seven-lag model is exploratory and should not be used to assign independent effects to individual lags. Finally, the individual-level partial R² was small (0.15%), and the patient-level analysis does not eliminate the ecological limitation because exposure remained identical within calendar month.

## Conclusions

This 12-year, 633,838-measurement study identified a reproducible seasonal pattern in HbA1c that aligned most strongly with a climatological temperature series shifted by 2 months, with significant correspondence also at lags 1 and 3 months. The findings are consistent with previously reported seasonal variation in HbA1c, but the use of fixed monthly temperature normals means that an independent causal temperature effect and a specific biological 2-month delay cannot be established. The population-level seasonal amplitude was 0.235%, whereas the individual-level partial R² was 0.15%. The findings are therefore best interpreted as evidence of seasonal structure in HbA1c and its correspondence with climatological temperature, with potential relevance to population surveillance and longitudinal laboratory interpretation rather than as an individual treatment correction.

## Data Availability

Upon reasonable request and execution of a data-sharing agreement, de-identified aggregate summary data generated and analyzed during this study will be provided by the corresponding author, in accordance with institutional privacy regulations. Requests to access the datasets should be directed to zjlkc@139.com.
