# Supplementary material for: Calendar month seasonality of HbA1c and its phase relationship with climatological temperature in 633,838 clinical measurements
Source: Front Endocrinol (Lausanne). 2026 Oct 7;17:1990386. doi: 10.3389/fendo.2026.1990386 (PMC13683058; doi:10.3389/fendo.2026.1990386)
Supplement: Supplementary file 1 [file SupplementaryFile1.docx]

**Supplementary Material**

Calendar Month Seasonality of HbA1c and Its Phase Relationship With Climatological Temperature in 633,838 Hospital Records

**Supplementary Table S1. Cross-correlation between the fixed climatological temperature series and monthly mean HbA1c by lag (full time series, n = 134–136 monthly observations)**

| Temperature lag | n | Pearson's r | 95% CI | P value | Spearman's ρ | P value |
| --- | --- | --- | --- | --- | --- | --- |
| Lag 0 (same month) | 136 | −0.127 | [−0.289, +0.044] | 0.140 | −0.106 | 0.235 |
| Lag 1 month | 135 | −0.249 | [−0.401, −0.084] | 0.004 | −0.231 | 0.007 |
| Lag 2 months ✦ | 134 | −0.297 | [−0.444, −0.134] | <0.001 | −0.291 | <0.001 |
| Lag 3 months | 133 | −0.264 | [−0.415, −0.098] | 0.002 | −0.267 | 0.002 |
| Lag 4 months | 132 | −0.167 | [−0.329, +0.004] | 0.056 | −0.174 | 0.046 |
| Lag 5 months | 131 | −0.035 | [−0.206, +0.137] | 0.690 | −0.054 | 0.539 |
| Lag 6 months | 130 | +0.105 | [−0.068, +0.273] | 0.233 | +0.084 | 0.344 |
| Seasonal (lag 2)* | 12 | −0.977 | [−0.994, −0.917] | <0.001 | — | — |

Note:Lag 2 months had the largest absolute Pearson correlation, but the lag 3 correlation was also significant and similar in magnitude. Because the temperature series consists of fixed monthly climatological normals repeated across years, the analysis supports a phase relationship over approximately 1 to 3 months rather than a uniquely identified biological delay. Newey West robust standard errors were used in regression models with a lag bandwidth of 6.

**Supplementary Table S2. Regression model results: ambient temperature (lag 2 months) and harmonic seasonality as predictors of monthly mean HbA1c**

| Model / Term | β | SE | 95% CI | P value | R² | n |
| --- | --- | --- | --- | --- | --- | --- |
| Model A — temperature lag 2 only |  |  |  |  | 0.0883 | 134 |
| Intercept | 7.1002 | 0.0484 | [7.005, 7.195] | <0.001 |  |  |
| Temperature (lag 2 months) | −0.00890 | 0.00249 | [−0.014, −0.004] | <0.001 |  |  |
| Model B — temperature lag 2 + harmonic seasonality |  |  |  |  | 0.1027 | 134 |
| Intercept | 6.9831 | 0.0553 | [6.874, 7.092] | <0.001 |  |  |
| Temperature (lag 2 months) | −0.00862 | 0.00138 | [−0.011, −0.006] | <0.001 |  |  |
| sin(2π·month/12) | −0.0512 | 0.0201 | [−0.091, −0.011] | 0.013 |  |  |
| cos(2π·month/12) | −0.0892 | 0.0183 | [−0.126, −0.053] | <0.001 |  |  |
| Seasonal regression (n=12 calendar months) |  |  |  |  | 0.9548 | 12 |
| Intercept (seasonal) | 7.1445 | 0.0312 | [7.074, 7.215] | <0.001 |  |  |
| Temperature (lag 2 months) | −0.01037 | 0.00072 | [−0.0120, −0.0088] | <0.001 |  |  |

Note:All models use Newey–West heteroscedasticity- and autocorrelation-consistent standard errors (lag = 6). Seasonal regression: n = 12 calendar-month means pooled across all years.

Supplementary Figure S1 | Full time series co variation of monthly mean HbA1c and the climatological temperature series from 2014 to 2025. The temperature line represents fixed monthly normals rather than year specific observations. The inverse seasonal co variation is shown for descriptive purposes.

Supplementary Figure S2 | Age stratified calendar month HbA1c pattern and phase relationship with climatological temperature. Panel a shows monthly mean HbA1c by calendar month pooled across 2014 to 2025. Panel b shows Pearson correlation for the 2 month phase offset by age group.
